# In search of female sterility causes in the tetraploid and pentaploid cytotype of *Pilosella brzovecensis* (Asteraceae)

**DOI:** 10.1007/s10265-021-01290-8

**Published:** 2021-04-03

**Authors:** Agnieszka Barbara Janas, Zbigniew Szeląg, Krystyna Musiał

**Affiliations:** 1 Department of Plant Cytology and Embryology, Institute of Botany, Jagiellonian University, Gronostajowa 9, 30-387 Cracow, Poland; 2 The Franciszek Górski Institute of Plant Physiology, Polish Academy of Sciences, Niezapominajek 21, 30-239 Cracow, Poland; 3 Institute of Biology, Pedagogical University of Cracow, Podchorążych 2, 30-084 Cracow, Poland

**Keywords:** Apomixis, Callose, Hawkweed, *Hieracium*, Ovule, Reproduction

## Abstract

Within the agamic *Pilosella* complex, apomixis (asexual reproduction through seed) involves apospory, parthenogenesis, and autonomous endosperm development. Observations of reproductive biology in *P. brzovecensis* throughout four growing seasons in the garden have shown that both tetraploid and pentaploid plants of this species do not produce viable seeds and reproduce exclusively vegetatively by underground stolons. The reasons for the seed development failure were unknown, therefore our research focused on the analysis of reproductive events in the ovules of this taxon. We found that apospory was initiated in the ovules of both cytotypes. Multiple aposporous initial (AI) cells differentiated in close proximity to the megaspore mother cell (MMC) and suppressed megasporogenesis at the stage of early prophase I. However, none of the AI cells was able to further develop into a multi-nucleate aposporous embryo sac (AES) due to the inhibition of mitotic divisions. It was unusual that callose was accumulated in the walls of AI cells and its synthesis was most likely associated with a response to the dysfunction of these cells. Callose is regarded as the isolating factor and its surprising deposition in the ovules of *P. brzovecensis* may signal disruption of reproductive processes that cause premature termination of the aposporous development pathway and ultimately lead to ovule sterility. The results of our embryological analysis may be the basis for undertaking advanced molecular studies aimed at fully understanding of the causes of female sterility in *P. brzovecensis*.

## Introduction

In Europe, *Hieracium* L. (*Hieracium* s. lat.) comprises two genera, *Hieracium* s. str. and *Pilosella* Vaill. In addition to the obvious morphological differences, both genera also differ in the mode of reproduction of apomictic species; mitotic diplospory of the *Antennaria* type in *Hieracium* s. str. and apospory in *Pilosella* (Asker and Jerling 1992; Gustafsson 1946; Nogler 1984). Linear megaspore tetrads in the ovules of amphimictic *Hieracium* and *Pilosella* species are, in turn, formed due to regular meiosis, and the reduced female gametophyte of the *Polygonum* type develops from the chalazal megaspore after three mitotic divisions (Koltunow et al. 1998; Pogan and Wcisło 1995). A common feature of *Hieracium* s. str. and *Pilosella* is relatively frequent intrageneric hybridization leading to the emergence of new, polyploid hybrids subsequently fixed by way of apomixis; this results in a large number of species with complicated taxonomic relations (Zahn 1930, 1935, 1938).

Both genera comprise polyploid complexes with a basic chromosome number x = 9. Within *Hieracium* s. str., diploids, triploids, tetraploids and very rare pentaploids are known (Musiał and Szeląg 2015, 2019). *Pilosella* shows a broad range of ploidy levels, i.e. from diploid to octoploid; moreover, its species are often represented by two or more cytotypes (Rotreklová et al. 2002, 2005; Schuhwerk and Lippert 1997). Sexual diploid species of *Hieracium* s. str. and *Pilosella* are rare and occur mainly in refugial regions of southern Europe (Merxmüller 1975) such as the Balkan Peninsula where diploid populations are consistently sought (Szeląg and Ilnicki 2011; Szeląg et al. 2007). Recently, we have focused on *Pilosella brzovecensis* (Horvat & Pawł.) Soják which belongs to *P*. sect. *Echininae* (Nägeli & Peter) Schljakov, earlier described from Northern Macedonia as *Hieracium brzovecense* Horvat & Pawł. by Pawłowski (1963). This morphologically outstanding mountain species, known only from a few relict sites on the Balkan Peninsula, has not yet been analysed karyologically. Four-year long observation of plants transferred to a garden has shown that *P*. *brzovecensis* does not produce fertile seeds; this could suggest that it reproduces sexually, however seed formation via cross fertilisation was prevented by the insufficient number of plants grown in the garden. Flow cytometry analysis revealed that the plants are tetraploid (2n ~ 4x) and pentaploid (2n ~ 5x), which could indicate the possibility of apomictic reproduction.

In light of this, we have undertaken research on developmental events in the ovules of this taxon. To date, embryological processes occurring in the ovules of female sterile *Pilosella* species have not been analyzed in detail. Specifically, for this reason, we believe that the results of our pioneer cytoembryological examination will contribute to clarifying the causes of ovule sterility in *P*. *brzovecensis* and potentially, in other *Pilosella* species that do not produce fertile seeds.

## Materials and methods

### Plant material

Living plants were collected in two disjoined localities in North Macedonia, on Mt. Ljuboten in the Šarplanina Mountains and on Mt. Solunska Glava in the Jakupica Mountains, and cultivated in an experimental garden of the second author. Voucher specimens are deposited in the herbarium of the W. Szafer Institute of Botany of the Polish Academy of Sciences (KRAM). A portion of the living plants collected in the field were also cultivated in the experimental garden of the Institute of Biodiversity and Ecosystem Research in Sofia and checked for ploidy level by V. Vladimirov following the method described in Szeląg and Vladimirov (2019).

For embryological studies, the inflorescences (capitula) at various developmental stages (2.5–6 mm long) were fixed in a mixture of glacial acetic acid and absolute ethanol (1:3, v/v) for at least 24 h. Then the plant material was transferred to 70 % ethanol and stored at 4 °C. The number of analysed inflorescences and florets is presented in Table 1.

**Table 1:** Numerical data on the *Pilosella brzovecensis* plant material used in this study and the incidence of ovules with multiple aposporous initial cells (AICs)

Cytotype of *P. brzovecensis*	No of capitula sampled	No of florets analysed	No of ovules with multiple AICs	Frequency of multiple AICs (%)
4x	14	104	59	56.7
5x	17	172	155	90.1

### Tissue clearing technique

Florets were isolated from fixed capitula, dehydrated in a graded ethanol series (70–100 %) and incubated in solutions of absolute ethanol and methyl salicylate (Sigma-Aldrich) (3:1, 1:1, 1:3, v/v) and in two changes of pure methyl salicylate according to protocol described by Musiał et al. (2015). The cleared plant material was analysed under a Nikon Eclipse 80i microscope fitted with Nomarski’s interference contrast (DIC optics).

### Histological analysis

Whole capitula or isolated florets were dehydrated in a graded ethanol series (70–100 %), infiltrated in mixtures of absolute ethanol and chloroform (3:1, 1:1, 1:3 v/v) for 1 h in each. Then samples were kept at 56 °C in pure chloroform with the addition of paraffin and after embedding in pure paraffin, they were cut into 7 μm thick sections which were stained with Heidenhain’s haematoxylin with Alcian Blue 8GX, mounted in Entellan (Merck) and examined using a Nikon 400 Eclipse microscope.

### Determination of pollen stainability (viability)

The viability of pollen grains was determined using the acetocarmine test in which the cytoplasm turns red in viable pollen and remains transparent in non-viable pollen (Singh 2003). The anthers were excised from the fixed florets and placed on a slide in a drop of 1% acetocarmine, covered with a coverslip and squashed. The samples were examined using a Nikon Eclipse TS100 microscope and image analysis software (NIS Elements D). Over 500 pollen grains from each cytotype were scrutinised.

### Detection of callose

The procedure for preparing the plant material was as previously described by Musiał and Kościńska-Pająk (2017, 2019). Florets were isolated from capitula and placed in 80 % ethanol for 1 h. Then the plant material was softened in 1 N NaOH at 37 °C for 4 h. Samples were rinsed with distilled water three times and once with 0.1 M K_3_PO_4_, stained overnight at room temperature with decolorized aniline blue (DAB; 0.1 % w/v). After rinsing with 0.1 M K_3_PO_4_, florets were placed on a microscope slide in a drop of K_3_PO_4_ mixed with a drop of glycerol. Subsequently, the ovules were isolated from ovaries and squashed under a cover slip. The samples were observed under UV light using a Nikon Eclipse E400 microscope fitted with an Epi-Fl Filter Block N UV-2 A which consists of an excitation filter EX330–380, a dichroic mirror DM400 and a barrier filter BA420.

## Results

### Reproductive processes in anthers

**Fig. 1:**
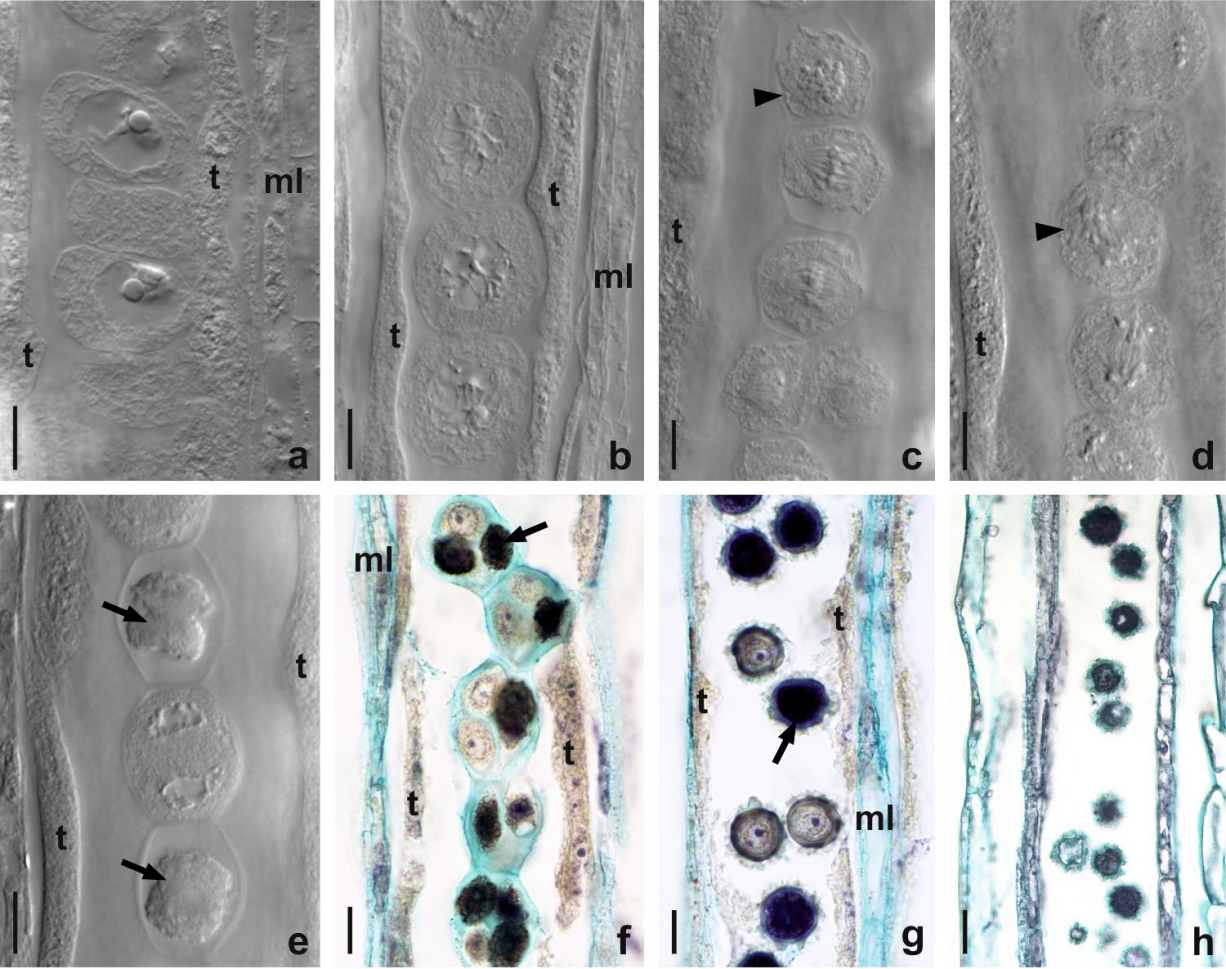
Developmental events in anthers of *P. brzovecensis*. Images obtained from cleared florets using Nomarski DIC optics (**a**–**e**) and longitudinal sections of anthers (**f**–**h**). **a**,** b** Prophase I in pollen mother cells (PMC). **c** Metaphase I in PMC, *arrowhead* indicates irregular metaphase plate. **d** Disturbed anaphase I in PMC, laggard chromosomes visible (*arrowhead*). **e** Microsporocytes at the interkinesis stage, *arrows* point to degenerating microspore dyads. **f** Microspore tetrads, note cell degeneration (*arrow*). **g** Anther at the free microspores stage, *arrow* shows degenerated microspore. **h** Older anther containing variable-size microspores, numerous cells without cytoplasm visible. Abbreviations: *ml* middle layer, *t* tapetum. Scale bars: **a**–**g** 10 μm, **h** 25 μm

The inflorescence of *P. brzovecensis* comprises only hermaphroditic, ligulate florets. In young florets of both cytotypes, anthers were properly formed and individual anther loculi were filled with sporogenous tissue surrounded by a fully developed anther wall consisting of four layers: epidermis, endothecium, middle layer, and tapetum. Microscopic examination of pollen mother cells (PMC) in tetraploid and pentaploid plants revealed an irregular microsporogenesis in both cytotypes (Fig. 1a–f). The disorders observed during the first meiotic division most often related to the occurrence of univalent, laggard chromosomes and the degeneration of numerous dyads of microspores (Fig. 1c–e). After completion of meiosis, tetrads of microspores were formed, however, most microspores within tetrads showed signs of cell degeneration (Fig. 1f). In older anthers, only a few viable microspores were observed, while the remaining microspores had no cell content (Fig. 1g, h). Furthermore, a clear differentiation in microspore size was visible (Fig. 1h). The acetocarmine test showed markedly reduced pollen stainability (viability) in *P. brzovecensis*. Based on the test results, we estimated it to be 38.60 and 30.22 % in the tetraploid and pentaploid cytotype, respectively.

### Reproductive processes in ovules

**Fig. 2:**
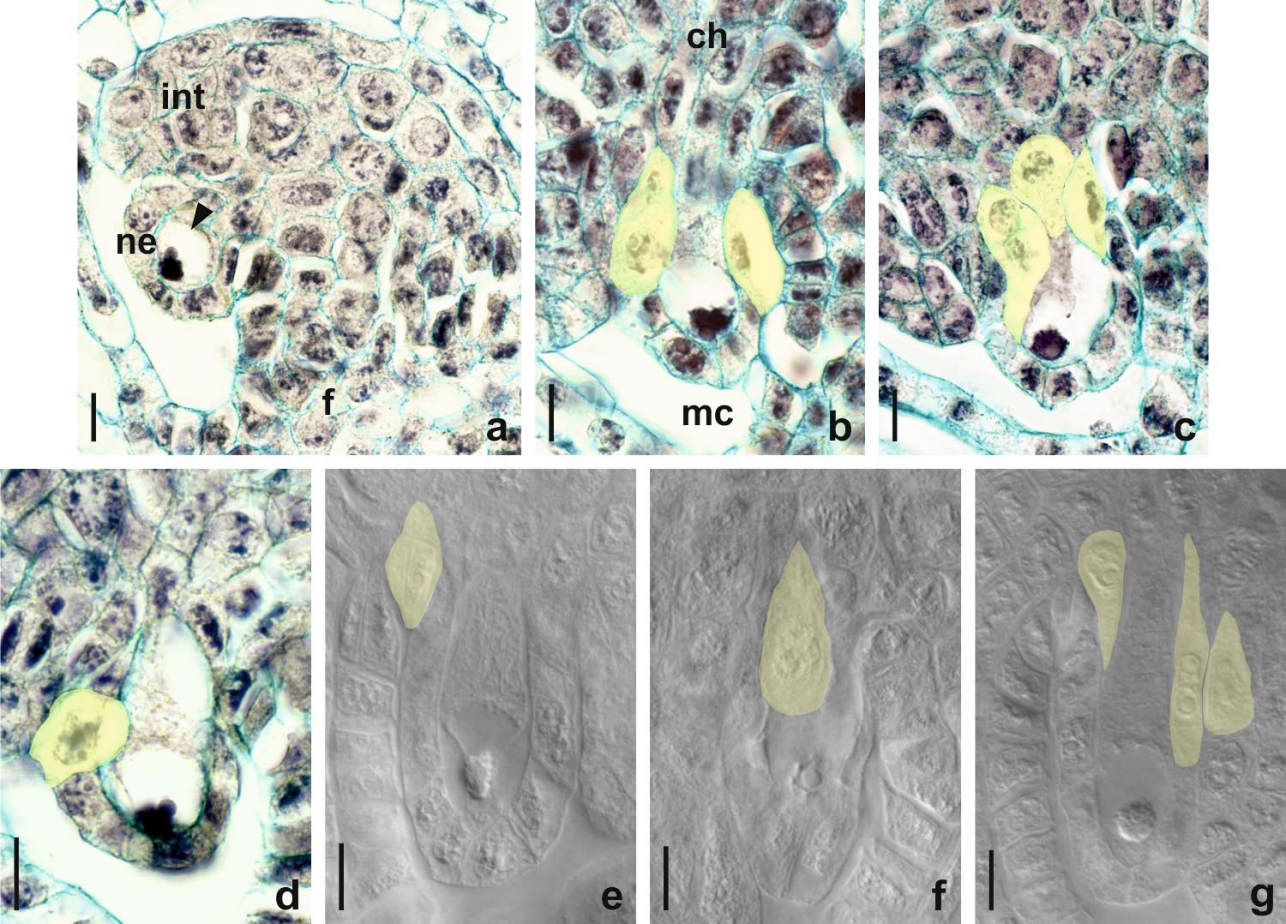
Early reproductive processes in ovules of *P. brzovecensis*. Longitudinal sections of ovules (**a–d**) and images obtained from cleared ovaries using Nomarski DIC optics (**e–g**). **a** Young ovule in the pre-meiotic stage, archesporial cell visible (*arrowhead*). **b–g** Micropylar region of ovules with a visible MMC at early prophase I stage and AI cells (*yellow shaded*) differentiating in its immediate vicinity. *ch* chalaza, *f* funiculus, *int* integument, *mc* micropylar canal, *ne* nucellar epidermis. Scale bars: 10 μm

**Fig. 3:**
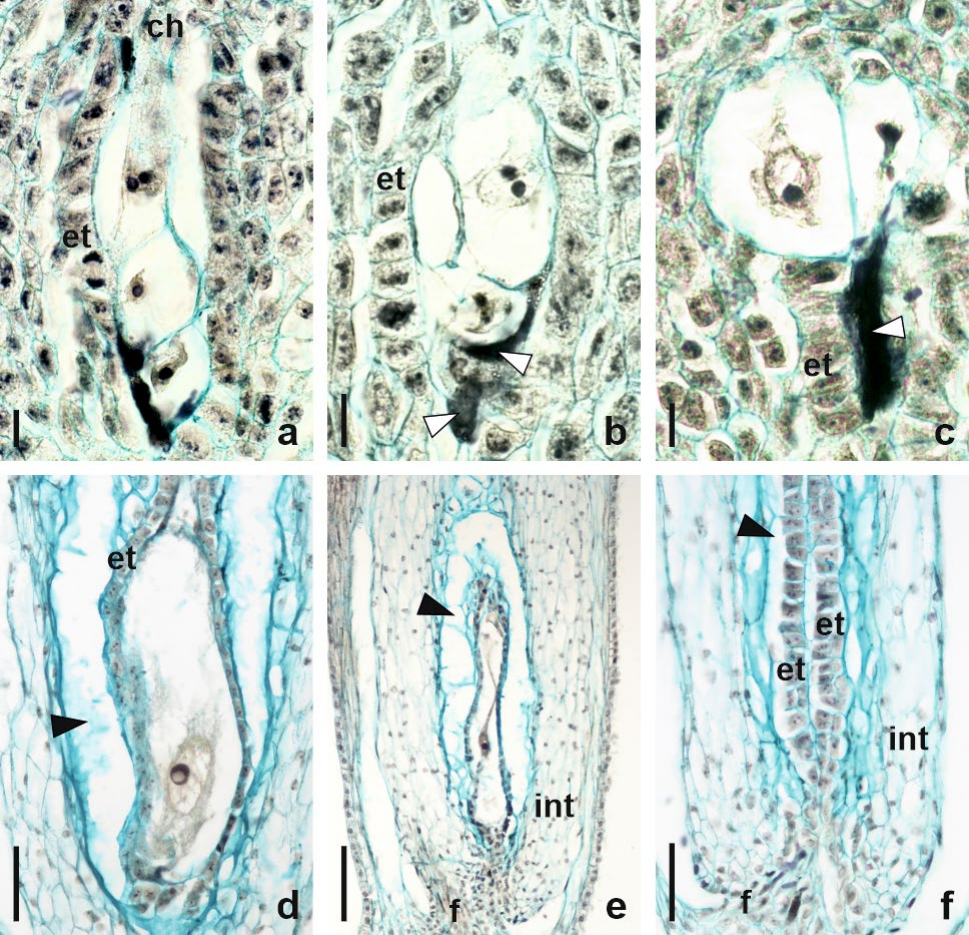
Developmental events in older ovules of *P. brzovecensis*. **a**,** b** Several AESs surrounded by endothelium, *arrowheads* indicate residues of degenerated cells at the micropylar pole. **c** Two AESs located deeper in the chalaza, *arrowhead* points to traces of degenerated cells in the endothelium region. **d** Abnormally enlarged one-nucleate embryo sac in mature ovule, *arrowhead* indicates liquefied peri-endothelial zone of integument. **e** Mature anatropous ovule with collapsing one-nucleate embryo sac, liquefied zone of integument visible (*arrowhead*). **f** Mature ovule with collapsed endothelium and without embryo sac, *arrowhead* shows peri-endothelial zone. *ch* chalaza, *et* endothelium, *f* funiculus, *int* integument. Scale bars*:* **a–c** 10 μm; **d** 50 μm; **e, f** 100 μm

An unilocular ovary of a bicarpellate gynoecium had a basal placentation and one anatropous, tenuinucellate, and unitegmic ovule. At a very early stage of ovule development, a single archesporial cell differentiated just below the epidermis of the nucellus. This strongly enlarged cell had a dense cytoplasm and a large nucleus with apparent nucleolus (Fig. 2a). The archesporial cell gradually elongated in the micropylar-chalazal axis and transformed directly into a MMC entering the prophase I of meiotic division. At this stage, the formation of one or more AI cells was observed in the ovules of both analysed cytotypes (Fig. 2b–d). The differentiation of somatic cells into AI cells was accompanied by a change in morphological features. The enlarged AI cells were vacuolated and usually took the shape of a drop or spindle (Fig. 2b–g). In the analysed ovules, we most often observed three to six AI cells that were differentiated in the immediate vicinity of the MMC. Out of 104 investigated ovules of the tetraploid cytotype of *P. brzovecensis*, multiple AI cells were found in 59 ovules, while in the pentaploid cytotype in 155 out of the 172 analysed (Table 1). AI cells were usually located close to the MMC chalazal pole (Fig. 2b, c, e–g), but in rare cases they also formed in the micropylar region from epidermal cells of the nucellus (Fig. 2d). In the ovules of both analysed cytotypes of *P. brzovecensis*, coexistence of the MMC and AI cells was temporary due to the very expansive growth of all AI cells that suppressed and completely destroyed the sexual pathway and eventually replaced the degenerate MMC. We did not observe either megaspore dyads or tetrads in any of the analysed young ovules. In older ovules, some of the AI cells located in the micropylar region also degenerated and their remnants along with traces of degraded sexual cells were visible below significantly expanded AESs (Fig. 3a–c). The observed one-nucleate AESs were situated linearly or lying side by side and characterized by a strong vacuolization of the cytoplasm and a clearly enlarged nucleus (Fig. 3a–c). In some ovules, they were surrounded by a layer of endothelium (Fig. 3a, b), but AESs located deeper in the chalaza were also frequently noticed (Fig. 3c). Our observations indicated that the AESs did not undergo mitotic divisions and their further development was limited only to enlargement. Hence, fully developed ovules usually contained one very elongated one-nucleate AES that gradually collapsed and the ovules became completely sterile (Fig. 3d–f). The ovules containing abnormally increased one-nucleate or collapsed embryo sac had well-developed endothelium and peri-endothelial tissue (Fig. 3d–f). In the analyzed material we did not observe formed multi-nucleate female gametophytes as well as the presence of embryos and endosperm.

**Fig. 4:**
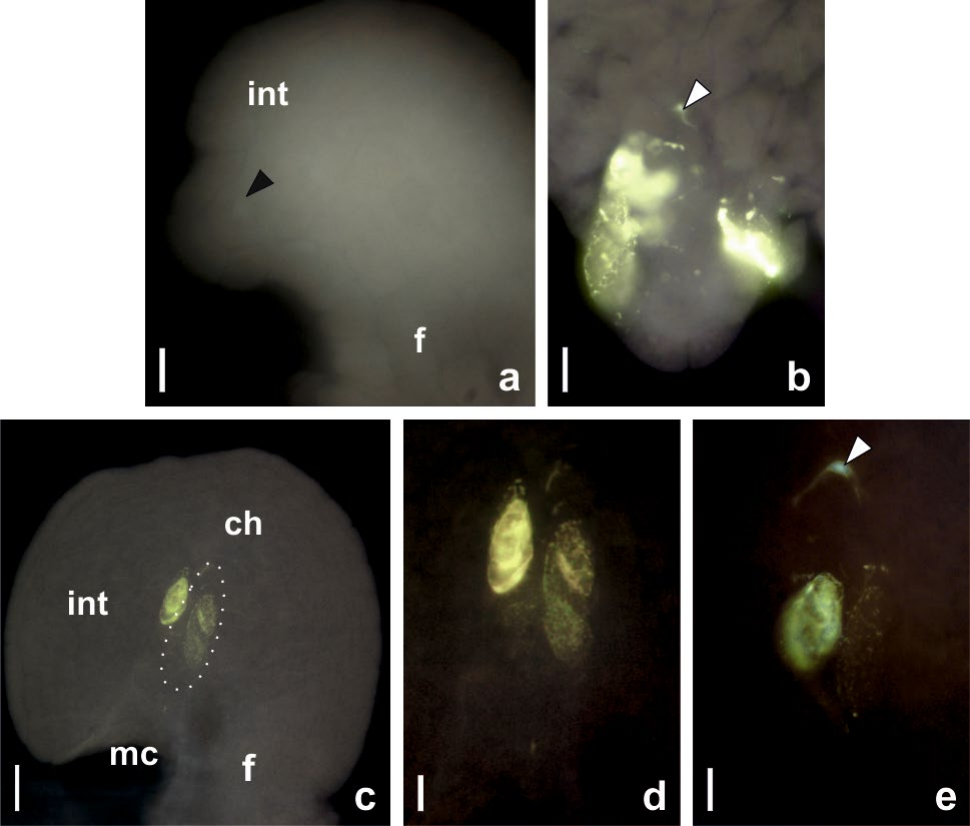
Callose localization in ovules of *P. brzovecensis.* **a** Young ovule with archesporial cell (*arrowhead*) whose wall is devoid of callose. **b** MMC and differentiating AI cells in its vicinity; callose deposits noticeable at the chalazal pole of the MMC (*arrowhead*) and strong fluorescence of callose evident in walls of the AI cells. **c** Anatropous ovule with a visible several AI cells containing callose in their walls; the MMC position marked with *dashed line*. **d** Magnification of the micropylar ovule region from **c**; note the differences in the intensity of the fluorescent signal indicating the variable content of callose in the walls of individual AI cells. **e** MMC with pronounced deposits of callose at the chalazal pole (*arrowhead*) and two AI cells significantly differing in content of callose in the cell walls. Abbreviations: *ch* chalaza, *f* funiculus, *int* integument, *mc* micropylar canal. Scale bars: **a**, **b**, **d**, **e** 10 μm; **c** 25 μm

It cannot be ruled out that the sterility of *P. brzovecensis* ovules is associated with atypical callose deposition in cell walls that we observed during early reproductive processes. As a rule, callose is a marker of megasporocyte wall, while the walls of cells initiating aposporous development do not contain callose. Our results of aniline blue staining confirmed that the wall of the archesporial cell was free of callose (Fig. 4a), but callose appeared in the wall of the MMC after the initiation of prophase I and was especially visible at the chalazal pole of the cell (Fig. 4b, d, e). Unexpectedly, a very strong fluorescent signal disclosing the presence of callose was also visible in the walls of all forming AI cells (Fig. 4b–e). The varied intensity of the observed fluorescent signal indicated that the walls of individual AI cells differed in callose content (Fig. 4c–e). We established that AI cells whose walls showed a higher level of callose accumulation were degraded at the earlier developmental stages. Our observations may suggest that this unusual accumulation of callose in the walls of AI cells was involved in arresting reproductive development at an early stage and, as a consequence, contributed to the sterility of ovules in the analysed tetraploid and pentaploid plants of *P. brzovecensis*. As a result of these disorders, the development of female gametophytes and then embryos and endosperm were not possible and the achenes of this species did not contain seeds.

## Discussion

Among angiosperm families, apomixis is most widespread in the Poaceae, Rosaceae, and Asteraceae (Asker and Jerling 1992; Bicknell and Koltunow 2004; Noyes 2007; Ozias-Akins 2006). In most apomictic plants, the formation of viable seeds depends on the presence of pollen as fertilization of the central cell is required for endosperm development (pseudogamy); however, in some apomictic taxa found mainly in the Asteraceae family the endosperm can develop autonomously (Hand and Koltunow 2014; Hörandl 2010; Koltunow and Grossniklaus 2003; Nogler 1984; Noyes 2007). Apomictic *Pilosella* species produce seeds by apospory coupled with fertilization-independent formation of an embryo and endosperm (Koltunow 2000; Koltunow et al. 1995, 1998; Pogan and Wcisło 1995; Skalińska 1971, 1973). Genetic and molecular analyses of apomictic reproduction basis in *Pilosella* have shown that components of apomixis are controlled by three dominant independent genetic loci. Namely, the *LOSS OF APOMEIOSIS* (*LOA*) locus stimulates the differentiation of somatic AI cells close to sexually programmed cells and regulates mitotic development of unreduced AES, the *LOSS OF PARTHENOGENESIS* (*LOP*) locus is required for both autonomous embryo and endosperm development, and the *AutE* locus, which is genetically separable from *LOA* and parthenogenesis, controls autonomous endosperm formation (Catanach et al. 2006; Henderson et al. 2017; Koltunow et al. 2011b; Ogawa et al. 2013). It is common knowledge that sexual and apomictic modes of reproduction in *Pilosella* are closely interrelated and that the initiation of megasporogenesis is necessary to stimulate the function of the *LOA* locus as well as that the formation of the AES triggers the demise of meiosis-derived cells and the termination of the sexual pathway (Juranić et al. 2018; Koltunow et al. 2011a, b; Okada et al. 2013; Tucker et al. 2003). Notwithstanding, in some other aposporous apomicts, e.g. representatives of *Brachiaria* and *Paspalum* genera, the sexual and aposporous reproductive pathways can coexist in a single ovule which, consequently, can contain both reduced and unreduced female gametophytes (Araujo et al. 2005; Hojsgaard et al. 2008). In *Pilosella* apomicts, the meiotic development is suppressed at the stage of the MMC or upon the completion of meiosis at the stage of the functional megaspore (Koltunow 2000; Koltunow et al. 1998). Our observations of reproductive events in the ovules of *P. brzovecensis* revealed that apospory occurred in both tetraploid and pentaploid plants, even though some *Pilosella* tetraploids can be obligate amphimictic (Bräutigam and Bräutigam 1996). An example would be the tetraploid cytotype of *P. officinarum* which reproduces only sexually (Gadella 1987; Koltunow et al. 1998; Pogan and Wcisło 1995; Sak et al. 2016). In *P. brzovecensis* ovules, the sexual pathway was already suppressed at the early stage of the MMC and the degeneration of germline cell was accompanied by the differentiation of multiple AI cells. The number of developing AI cells may vary between apomictic *Pilosella* species. For example, only one AI cell was most frequently observed in the ovules of triploid *P. piloselloides*, whereas in the ovules of a naturally occurring aneuploid *P. aurantiaca* (2n = 3x + 4 = 31), up to eight AI cells developing into embryo sacs were detected (Koltunow et al. 1998). In the latter case, the number of embryo sacs decreased during further development and ultimately the older ovules contained a single mature female gametophyte only (Koltunow et al. 1998, 2000). Although AI cells as well as young AESs may apparently compete for space and nutrients inside the ovules of *P. brzovecensis*, this competition does not appear to be a major cause of complete female sterility in light of previous observations of reproductive processes in other *Pilosella* apomicts. An intriguing observation was that in the ovules of *P. brzovecensis* plants, callose was present not only in the wall of the MMC but it was also deposited in the walls of the AI cells. Callose is known to be a marker of sexually programmed cells in the ovules of many angiosperm plants (Rodkiewicz 1970). In contrast to this, the walls of AI cells do not contain callose, as previously found in aposporous *Poa pratensis* (Naumova et al. 1993), *Panicum maximum* (Naumova and Willemse 1995), *Pennisetum* sp. (Peel et al. 1997), *Brachiaria decumbens* (Dusi and Willemse 1999), and *Pilosella* sp. (Tucker et al. 2001). Similarly to the ovules of *P. brzovecensis*, callose accumulation in the walls of AI cells was characteristic of the experimentally induced *loa1* mutant *P. aurantiaca* which largely lost the ability to produce apomictic seed due to a defect in the formation of functional AI cells (Okada et al. 2007). It is believed that in apomictic *Pilosella*, contact of AI cells with sexually programmed cells is required to initiate AI mitosis and the degeneration of sexual cells (Juranić et al. 2018). On the contrary, in *loa1* mutant, the AI cells differentiated and developed at some distance from the MMC, so it was suggested that the alternative positioning and growth direction of these cells may affect cell-cell communication and result in defective signaling from adjacent cells, which prevents mitotic division and the development of AESs (Okada et al. 2007). In the case of *P. brzovecensis*, AI cells appeared in close proximity to the MMC but did not develop into multi-nucleate embryo sacs. It cannot be ruled out that callose accumulation in the walls of AI cells was a response to their dysfunction. It should be emphasized that callose deposition has been associated with ovule sterility in different species and with different sterility mechanisms, and this may be seen as a manifestation of genetic determination of female sterility (Byzova et al. 1999; Dumas and Knox 1983; Mól et al. 2011; Rosellini et al. 1998, 2003; Sun et al. 2004; Vishnyakova 1991). Therefore, it is likely that the synthesis of callose in the walls of AI cells was the first indication of abnormal reproductive development leading to female sterility of *P. brzovecensis* plants. It should be noted that the ovules of both cytotypes were properly developed and displayed a typical structure of the integument with a distinguished endothelium and a peri-endothelial zone whose cells have undergone liquefaction. A liquefaction (or gelatinization) of the integument cells adjacent to endothelium was also observed in other sexual and apomictic *Pilosella* species (Koltunow et al. 1998; Płachno et al. 2017). Similarly, the peri-endothelial zone occurrence was characteristic of the ovules of *Taraxacum* and *Chondrilla* species (Cooper and Brink 1949; Janas et al. 2016; Musiał and Kościńska-Pająk 2013; Musiał et al. 2013; Płachno et al. 2016). The structural changes recorded in this region of the integument were accompanied by the accumulation of carbohydrate-rich material that can potentially be the source of nutrients for the developing female gametophyte and embryo (Cooper and Brink 1949; Koltunow et al. 1998; Musiał and Kościńska-Pająk 2013; Musiał et al. 2013; Płachno et al. 2016, 2017). It has also been suggested that the presence of this nutritive tissue may have facilitated the evolution of the autonomous embryo development in apomicts of the Asteraceae family (Koltunow et al. 1998; Van Baarlen et al. 1999).

## Conclusions

Our cytoembryological research has demonstrated that apospory is initiated in the ovules of both the tetraploid and pentaploid cytotype of *P. brzovecensis*; however, abnormal callose deposition in the walls of AI cells most likely contributed to premature termination of the aposporous reproductive pathway, which eventually led to seed set failure in the plants of this taxon. At this stage, we are not in a position to comprehensively explain the role of callose deposited in the walls of AI cells that differentiate in the ovules of *P. brzovecensis*. Therefore, it seems desirable to conduct further molecular analyses of the expression of genes that control apomictic development and regulate intercellular signaling pathways in the ovules of this taxon.
